# Preparation and Properties of Flexible Multilayered Transparent Conductive Films on Substrate with High Surface Roughness

**DOI:** 10.3390/ma18143389

**Published:** 2025-07-19

**Authors:** Mengfan Li, Kai Tao, Jinghan Lu, Shenyue Xu, Yuanyuan Sun, Yaman Chen, Zhiyong Liu

**Affiliations:** 1 School of Materials Science and Engineering, Henan Institute of Technology, Xinxiang 453003, China; 2 Engineering Research Center for Metallic Materials Modification Technology of Henan Province, Henan Institute of Technology, Xinxiang 453003, China; 3 Henan Key Laboratory of Advanced Cable Materials and Intelligent Manufacturing, Henan Institute of Technology, Xinxiang 453003, China; 4 National Materials Corrosion and Protection Scientific Data Center, University of Science and Technology Beijing, Beijing 100083, China

**Keywords:** multilayered structure, TCF, flexible, substrate, surface roughness, magnetron sputtering

## Abstract

The flexible transparent conductive films (TCFs) of a ZnS/Cu/Ag/TiO_2_ multilayered structure were deposited on a flexible PET substrate with high surface roughness using magnetic sputtering, and the effects of structural characteristics on the performance of the films were analyzed. The TCFs with TiO_2_/Cu/Ag/TiO_2_ and ZnS/Cu/Ag/ZnS symmetric structures were also prepared for comparison. The TCF samples were deposited using ZnS, TiO_2_, Cu and Ag targets, and they were analyzed using scanning electronic microscopy, atomic force microscopy, grazing incidence X-ray diffraction, spectrophotometry and a four-probe tester. The TCFs exhibit generally uniform surface morphology, excellent light transmittance and electrical conductivity with optimized structure. The optimal values are 84.40%, 5.52 Ω/sq and 33.19 × 10^−3^ Ω^−1^ for the transmittance, sheet resistance and figure of merit, respectively, in the visible spectrum. The satisfactory properties of the asymmetric multilayered TCF deposited on a rough-surface substrate should be mainly attributed to the optimized structure parameters and reasonable interfacial compatibilities.

## 1. Introduction

The transparent conductive film (TCF) is widely used as an important photoelectric material in fields such as flat panel displays, photovoltaic power generation, light-emitting diodes, transparent windows against electromagnetic interference and low-radiation glass [1,2,3]. The recent development of flexible electronic technology has been further expanding the application scenarios of the TCF [4,5,6,7,8,9]. Transparent conductive oxide (TCO) is the most widely used class of TCF materials [4,10]. However, due to the limitation on light transmittance, electric conductivity or mechanical flexibility, the introduction of highly conductive material such as nano silver wire, nano carbon tubes, graphene, metal grating or ultra-thin metal film (UTM) was studied to form composited TCF, with enhanced combination properties. Among them, the multilayered TCF with UTM as the middle layer reveals good light transmission, electrical conductivity, flexibility and customizability, showing great potential for engineering application [4,5]. Furthermore, its preparation process can meet the technical requirements of roll-to-roll (RTR) manufacturing [11].

The multilayer TCF usually exhibits a dielectric/metal/dielectric structure (D/M/D). Its electrically conductive properties mainly depend on the intermediate metallic layer, which possesses good performance.

The dielectric layers on both sides can significantly mitigate optical losses by concurrently suppressing broadband reflection from the metallic interlayer and quenching interfacial surface plasmon resonance (SPR) phenomena [11,12,13,14], so that the multilayered TCF has good light transmission in the visible light spectrum. The top dielectric layer could also provide protection for the active metallic layer by isolating the corrosive medium and improve the stability of the film [15]. What’s more, the composite film could maintain good electrical conductivity and certain functionality even when the dielectric layer is bent and broken due to the good flexibility of the metallic layer [9,10,16].

The flexible transparent polymer sheet/films (PET, PEN, PI, et al.) are usually adopted as the substrate material for flexible multilayered TCF. The original surface roughness ($R_{a}$) of the polymer film is mostly a few nanometers to tens of nanometers [17]. Since the lower substrate roughness (e.g., $R_{a}$ < 1 nm) can facilitate the uniformity, light transmission and electrical conductivity of the TCF, a smoothing coating is usually synthesized on the surface of the polymer film to alleviate the convex and concave fluctuation of the morphology. Dielectric materials with high transparency are usually selected as smoothing coating materials, such as NaF, LiF, SiO_2_, or acrylic resins, polyurethane resins, siloxane condensates [17], or a mixture of inorganic and organic substances [18]. The smoothing coating can be prepared by either dry coating methods, such as vacuum evaporation, or the wet coating method, which is the more popular way at present. The above methods of preparing the smoothing coating require high process accuracy and a long cycle, which increases the cost of the product, and is prone to quality defects such as abnormal elongation, folding, warping and uneven thickness. Besides the intrinsic surface roughness, the substrate morphology can be processed before the formation of TCF by corona discharge, ultraviolet irradiation or plasma etching, so as to improve the bonding between the substrate and deposited layers [11,18]. The pre-treatment may further increase the surface roughness of the substrate. These problems could be largely avoided if high-performance TCFs can be prepared directly on the polymer substrate without the smoothing coating.

ZnS is a widely utilized material for films and semiconductors, prized for its processability and cost-effectiveness. It exhibits a high refractive index (n ≈ 2.4) and excellent light transmittance in the visible spectrum, along with a wide bandgap (~3.6 eV) [19]. Compared with the other dielectric materials, ZnS demonstrates superior percolation properties toward intermediate metallic layer [16,20], facilitating the formation of continuous and uniform films at minimal thicknesses. When ZnS is selected as the bottom dielectric layer in the multilayered TCF, it will not only contribute to the optic performance but also improve the interfacial morphology between the substrate and sublayers, enhancing the film stability.

Ag is the most prevalent choice for the intermediate layer of multilayered TCFs due to its exceptional electrical conductivity, low light absorption coefficient, and strong plasmonic resonance effects [21,22]. During the initial deposition period, metallic materials tend to exhibit an island-like growth owing to its high surface energy, where nanoscale clusters form [23]. The metallic layer will not transition to a continuous film until it exceeds the critical thickness. Seed layers (e.g., Cu [24,25], Al [25,26], Ti [27,28]) can be employed to enhance the nucleation, lowering both critical thickness and surface roughness.

TiO_2_ as a semiconductor material possesses a wide bandgap (~3.0 eV), exhibiting a high refractive index (n ≈ 2.7) and over 90% light transmittance in the visible spectrum. It also boasts a high dielectric constant, high chemical stability, high photocatalytic activity, high moisture resistance, and high mechanical robustness. TiO_2_ film deposited via magnetron sputtering demonstrates excellent surface smoothness and compactness, making it ideal as the top dielectric layers.

The multilayered films fabricated via magnetron sputtering are studied in this paper, with the material group of ZnS-TiO_2_-Ag-Cu. The multilayer configurations have been rarely reported, especially with flexible substrate of high roughness. By optimizing the structural design and sputtering parameters, we aim to develop a high-performance flexible multilayered TCF on a cost-effective substrate with relatively high surface roughness.

## 2. Materials and Methods

Cu and Ag targets (diameter: 76.2 mm, purity ≥99.99%) were selected as raw materials for the intermediate metallic layers, while ZnS and TiO_2_ targets (≥99.99% purity) were used for the top and bottom dielectric layers. The commercial flexible PET sheets (supplied by Huanan Xiangcheng Technology Co. Ltd., Changsha, China) were employed as the substrate for TCF samples, with the thickness of 0.175 mm and planar dimensions of 20 × 20 mm. Prior to magnetron sputtering, the substrate underwent ultrasonic cleaning in acetone and anhydrous ethanol for 10 min each, followed by rinsing with deionized water and drying with high-purity N_2_ (≥99.99%).

A JSD-500 magnetron sputtering system (manufactured by Anhui Jiashuo Technology Co. Ltd., Hefei, China), equipped with four targets in a vacuum chamber (inner dimension 500 mm × 500 mm × 420 mm), was employed to fabricate the multilayered transparent conductive films. The dielectric layers were grown using radio frequency power sources (manufactured by Zhongshan Kaimei Electronics Co. Ltd., Zhongshan, China, model AG-1305 with power capacity of 500 W), TiO_2_ layer at 150 W and ZnS layer at 50 W. For the intermediate metallic interlayers, direct current power sources (manufactured by Shenyang Sciens Technology Co. Ltd., Shenyang, China, model DC500E with power capacity of 500 W) were utilized, Ag at 120 W and Cu at 10 W. The target-to-substrate distance was fixed at 90 mm. After evacuating the sputtering chamber to a base pressure of 5 × 10^−4^ Pa, the high-purity (≥99.999%) argon gas was injected at a flow rate of 70 sccm, and the chamber pressure was stabilized at 0.4 Pa by adjusting the baffle valve. All layers were sequentially deposited in the same vacuum chamber via pre-sputtering and formal sputtering, without external substrate heating. Under stable deposition conditions, layer thickness was managed by regulating the deposition duration. Deposition rates for each material were established beforehand (0.034 nm/s for ZnS, 0.047 nm/s for TiO_2_, 0.029 nm/s for Cu and 0.723 nm/s for Ag). The calibration involved measuring the thickness versus time using thicker reference films deposited under identical parameters. For the thin Cu layer with sub-nanometer scale thickness, which forms discontinuous island structures, the reported thickness represents an equivalent geometric thickness.

The microstructure of the deposited layers was examined using field-emission scanning electron microscopy (FE-SEM; Hitachi SU8010, Tokyo, Japan). Atomic force microscopy (AFM; Bruker Dimension Icon, Boston, MA, USA) was employed to evaluate the surface topography of non-conductive specimens. Phase identification was performed by grazing incidence X-ray diffraction (GIXRD; Rigaku Smart Lab, Tokyo, Japan) with a 2 °/min scanning speed. The relative light transmittance was determined by spectrophotometry (Youke T-UV756, Shanghai, China). In accordance with Equation (1),

(1) $$T_{av}=\frac{1}{n}\sum_{i=1}^{n}T_{i}$$

where $T_{i}$ is the transmittance value of a given wavelength, and n is the number of transmittance data in the defined light band. The average light transmittance ($T_{\mathrm{av}}$) was derived by calculating the arithmetic mean of the transmittance data across the relevant wavelength range. The electrical conductivity was evaluated by sheet resistance values using a four-point probe system (JG M-3, Suzhou, China). Non-contact profilometry (Rtec wl, San Jose, CA, USA) and spectroscopic ellipsometry (HORIBA SAS, Shanghai, China) were employed for the thickness measurements.

## 3. Results

### 3.1. Characterization of the Surface Morphology for the Substrate and Sublayers in the TCFs

**Figure 1:**
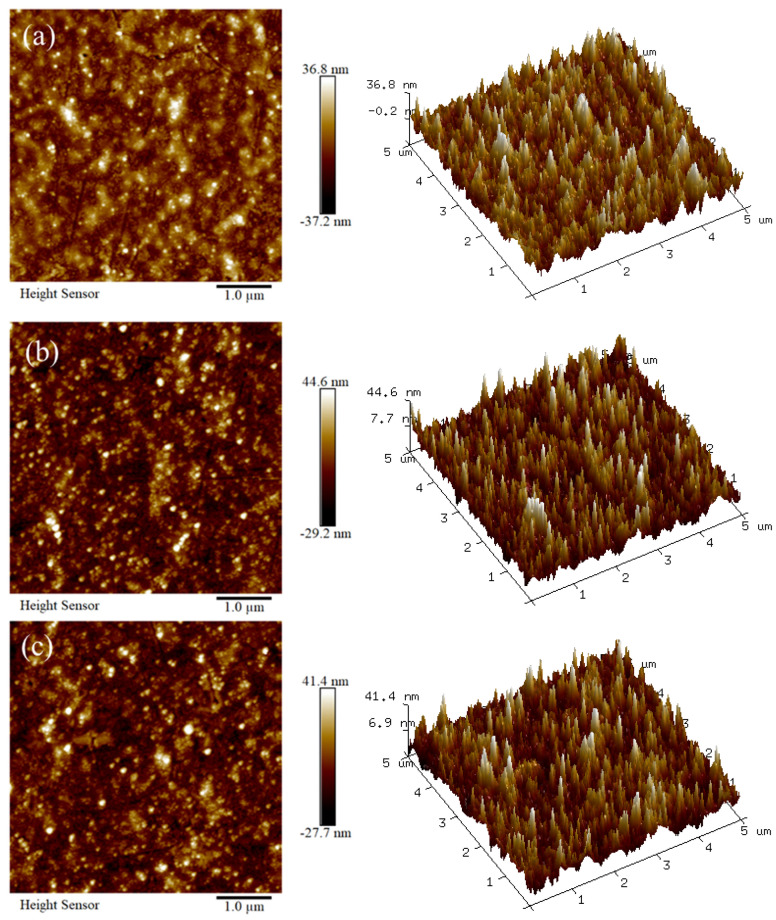
The surface topography (AFM) of the substrate and bottom layers: (**a**) PET; (**b**) PET/TiO_2_; (**c**) PET/ZnS.

AFM, a feasible way for non-conducting materials, was employed to probe the nanoscale topography of the PET substrate and bottom dielectric layers within the multilayered TCF architecture. The planar and three-dimensional images are shown in Figure 1. The original PET substrate exhibits a uniform surface morphology, as seen in Figure 1a, possessing a surface roughness of $R_{a}$ 6.66 nm. This roughness value is obviously larger than the popular flexible substrates with smoothing coatings (typically less than 1 nm) [17].

The TCF with a D/M/D structure is deposited sequentially, layer by layer. The bottom layer will be the first to be prepared. When the bottom layer forms on the surface of the PET substrate, its surface morphology changes with the varying dielectric materials and sputtering parameters. Figure 1b,c reveals the AFM pictures of the surface of TiO_2_ and ZnS as the bottom layer, respectively. The thickness values were selected as 35 nm and 40 nm, which are in the typical range of dielectric layer thickness. These images both display the homogeneous distribution of morphology features. The roughness values are $R_{a}$ 7.65 nm for the layer of TiO_2_ and $R_{a}$ 7.28 nm for ZnS. The surface roughness of the bottom layers slightly develops compared with the substrate.

**Figure 2:**
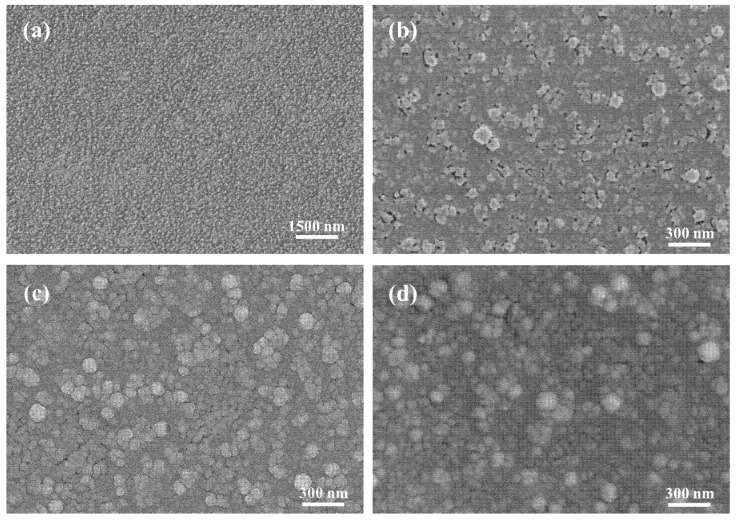
The surface morphology (SEM) of the multilayers with the bottom layer of TiO_2_: (**a**) PET/TiO_2_/Ag; (**b**) PET/TiO_2_/Cu/Ag; (**c**) PET/TiO_2_/Ag/TiO_2_; (**d**) PET/TiO_2_/Cu/Ag/TiO_2_.

The surface morphology of the intermediate and top layers for a multilayered TCF is analyzed using FE-SEM first with TiO_2_ as the bottom layer material, and the pictures are presented in Figure 2. A TiO_2_ layer with a thickness of 35 nm was sputtering deposited on the PET substrate, followed by an Ag layer with a thickness of 9.5 nm on the TiO_2_ layer, which corresponds to a film structure of PET/TiO_2_/Ag. The SEM photo of its surface morphology is presented in Figure 2a. Light colored Ag particles with a near-sphere shape are distributed in the TiO_2_ layer surface (dark area in the photo). The gaps between Ag particles accounts for a considerable area, which leads to a partially continuous Ag layer. This heterogeneous feature of the metallic layer provides poor electrical conductivity, which makes it difficult to obtain a clear high-magnification image (>10 k in second electron mode) during SEM analysis.

To facilitate the continuous forming of the Ag layer, the Cu seed layer was introduced into the multilayered film. A TiO_2_ bottom layer, a Cu seed layer and an Ag intermediate layer, with respective thickness of 35 nm, 0.25 nm and 9.5 nm, were sequentially deposited on the flexible substrate, corresponding to the film structure of PET/TiO_2_/Cu/Ag. The SEM picture is displayed in Figure 2b. The continuity and uniformity of the intermediate metallic layer is significantly improved compared with monolayer of Ag in Figure 2a. The bimetallic layer is continuous for most of the area, only with a small number of vacancies. Some metallic particles with a relatively large size of 70–130 nm are distributed evenly. The improved continuity of the metallic layer contributes the electrical conductivity, which makes it easy to acquire clear images in a high-magnification (50 k) mode.

The top dielectric layer was the last one to be deposited during the preparation of the multilayered TCF. The TiO_2_ layer with a thickness of 35 nm was magnetron sputtered on the above-mentioned two types of metallic layers, respectively. Then, the two complete D/M/D structures were obtained, which are PET/TiO_2_/Ag/TiO_2_ and PET/TiO_2_/Cu/Ag/TiO_2_. The SEM images for the surface morphology of these two samples are shown in Figure 2c,d. Although the intermediate layers possess diverse characteristics, as shown in Figure 2a,b, these two TCF samples exhibit similar top surface features, which are uniform and continuous, comprised by a large quantity of TiO_2_ particles with a relatively wide range of size distributions. It seems that the top dielectric layer with a certain thickness (e.g., 35 nm) could alleviate the morphology characters from the intermediate layer.

**Figure 3:**
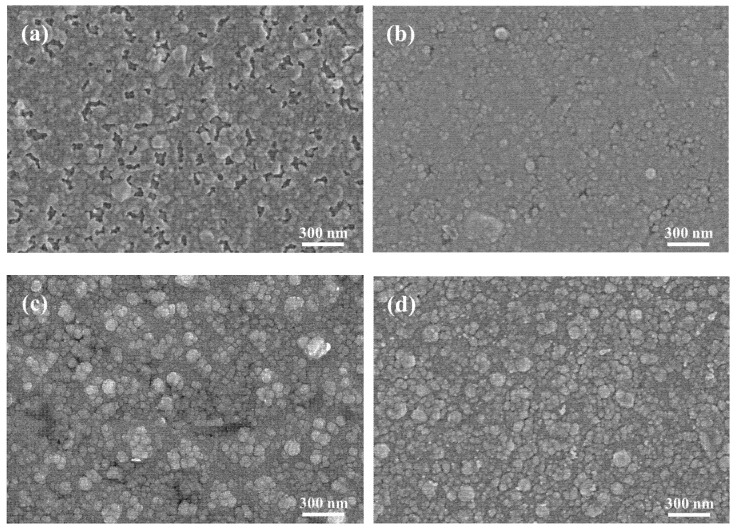
The surface morphology (SEM) of the multilayers with the bottom layer of ZnS: (**a**) PET/ZnS/Ag; (**b**) PET/ZnS/Cu/Ag; (**c**) PET/ZnS/Ag/ZnS; (**d**) PET/ZnS/Cu/Ag/ZnS.

The multilayered TCFs with ZnS as the bottom layer material was also characterized by FE-SEM in a stratified way. The bottom ZnS layer with a thickness of 40 nm was firstly sputtering deposited on the PET substrate, then a monolayer of Ag with a thickness of 9.5 nm was prepared on the ZnS layer, forming a bilayer structure of PET/ZnS/Ag. The SEM image of its surface is presented in Figure 3a, where most of the area is overlayed by Ag layer, with a few void areas where the ZnS layer of relatively dark color becomes visible. The surface profile of PET/ZnS/Ag is very similar to that of PET/TiO_2_/Cu/Ag, where the obvious discrepancy would be the larger percentage of the void zones.

The seed layer of Cu was also introduced as part of the intermediate layer for comparison. The ZnS layer (40 nm), Cu layer (0.25 nm) and Ag layer (9.5 nm) were sequentially deposited to form the multilayer of PET/ZnS/Cu/Ag, the SEM image of which is displayed in Figure 3b. This composite film possesses the best grade so far with respect to the completeness, homogeneity and smoothness. A fully continuous bimetal layer had formed on the bottom dielectric layer. Considering the factors of seed layer introduction and variation of bottom dielectric layer material, a conclusion could be drawn that the Cu seed layer improves the homogeneous nucleation of the Ag layer effectively, and the selection of the ZnS bottom layer facilitates the continuity and evenness of the intermediate layer, based on a flexible substrate with high surface roughness.

When further preparing the top dielectric layer on the metallic layers, the D/M/D structured TCFs with a ZnS bottom layer were acquired. The ZnS layers with a thickness of 40 nm were deposited on the Ag monolayer and Cu–Ag bilayer, respectively. The SEM images of the corresponding multilayers of PET/ZnS/Ag/ZnS and PET/ZnS/Cu/Ag/ZnS are presented in Figure 3c,d. Both the surfaces consist of ZnS particles with variated sizes. Relatively large-sized particles are distributed evenly in the flat matrix mainly comprised by small particles. The percentage of large particles in Figure 3d is higher than that shown in Figure 3c, perhaps due to the promotion effect on the layer growth from the smoother surface of the bimetallic layer.

**Figure 4:**
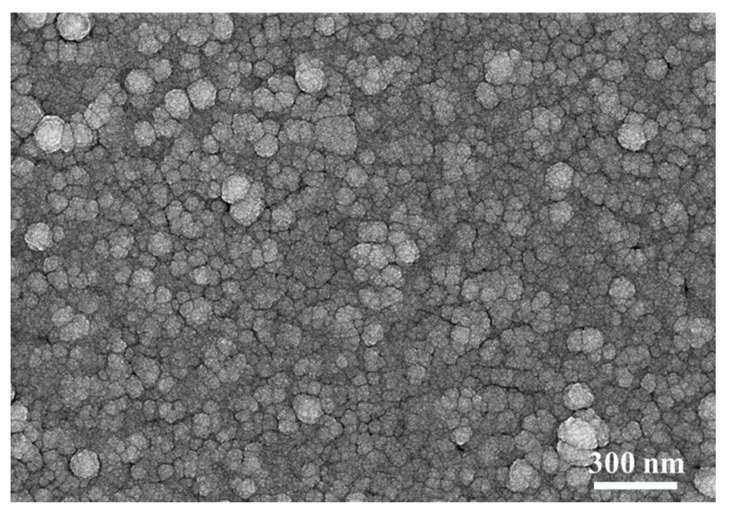
The surface morphology (SEM) of PET/ZnS/Cu/Ag/TiO_2_ multilayer.

Considering the excellent smoothing effect of the PET/ZnS/Cu/Ag structure, the TiO_2_ layer was then sputtering deposited on the top, aiming to combine its advantages of higher stability, mechanical properties and corrosion resistance for the multilayer TCF. Then, an asymmetric multilayered TCF with the structure of PET/ZnS/Cu/TiO_2_ was prepared; the thickness values of each layer are 41 nm, 0.25 nm, 9.5 nm and 38 nm, respectively. The SEM photo of its surface morphology is exhibited in Figure 4. The top layer is comprised by TiO_2_ particles, the profile features of which are similar to that shown in Figure 2c,d. The mild difference would be a narrower distribution range of the particle sizes, which could be attributed to a more equalized growth mode of the top layer on a smoother base.

### 3.2. Crystalline Phase Analysis of the Multilayered Film

**Figure 5:**
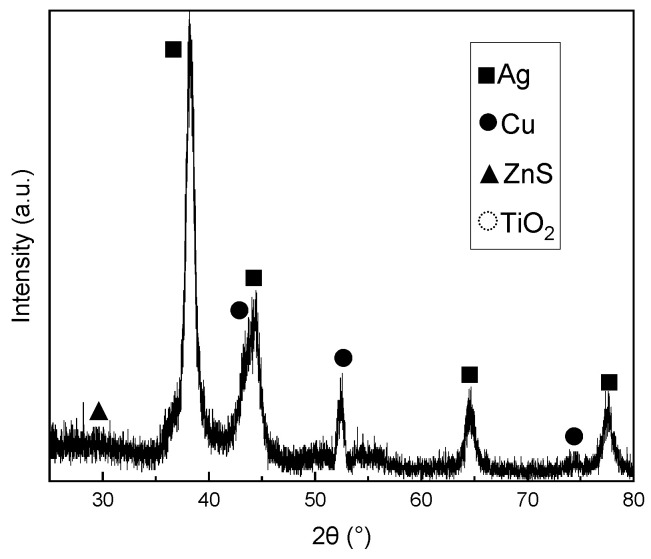
The GIXRD pattern of ZnS/Cu/Ag/TiO_2_ multilayer.

GIXRD was adopted for the crystalline phase analysis within the ZnS/Cu/Ag/TiO_2_ multilayer, which can overcome limitations associated with the ultrathin sublayer dimensions and significantly enhance the probe sensitivity to near-surface features. To further amplify effective diffraction signals, a designed large thickness scale of 40 nm was selected for each sublayer in the multilayer, with the structure abbreviated as ZnS40 Cu40 Ag40 TiO_2_40 (the following paper adopts the same abbreviation rule to name the multilayered samples). This specimen was fabricated under identical magnetron sputtering conditions. For GIXRD characterization, an optimized incidence angle of 1° was selected from the angular regime of 0.2–1.5°. Figure 5 reveals distinct diffraction features indexed to standard reference patterns. The dominant peak series originates from polycrystalline Ag (ICSD #67994), as annotated. Three additional well-defined peaks correspond to specific crystallographic planes of metallic Cu (ICSD #64699). A faint low-angle diffraction feature is attributable to the ZnS phase (ICSD #60378). Notably, no discernible TiO_2_-related diffraction peaks were detected. The GIXRD data indicate superior crystallinity in metallic layers (Ag, Cu) relative to dielectric layers. For the ZnS layer deposited without external heating [19,29], partial crystallization results in broadened diffraction peaks, as seen in Figure 4. Meanwhile, the TiO_2_ top layer retains an amorphous structure during formation [30,31,32], consistent with the absence of characteristic peaks.

### 3.3. The Optical Transmittance of Multilayered TCFs

**Figure 6:**
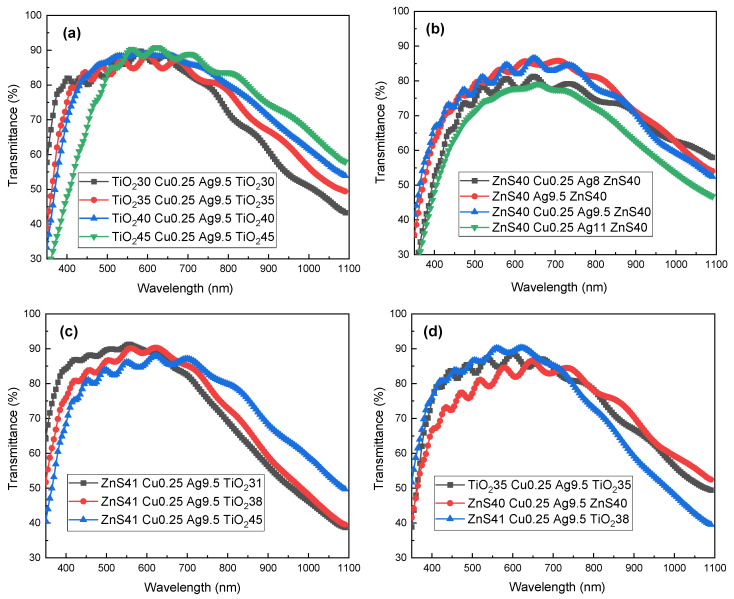
The light transmittance profiles of the multilayered TCFs: (**a**) PET/TiO_2_/Cu/Ag/TiO_2_; (**b**) PET/ZnS/Cu/Ag/ZnS; (**c**) PET/ZnS/Cu/Ag/TiO_2_; (**d**) contrast group.

The optical transmittance performance is analyzed for the multilayered TCFs. The transmittance curves for the films with PET/TiO_2_/Cu/Ag/TiO_2_ structure are shown in Figure 6a. The thickness value of the metallic bilayer for this TCF group was fixed at 9.5 nm for Ag and 0.25 nm for Cu. The thicknesses of the bottom and top dielectrics layer were adjusted synchronously from 30 nm to 45 nm, keeping a symmetric D/M/D structure. The transmittance variations in this group follow the similar trend as increasing rapidly to a maximum value and then decreasing steadily across the light wavelength range of 350–1100 nm. The peak values correspond to the wavelength of around 600 nm for all the four samples. With the thickness increase of the dielectric layers, the position of the transmittance curve moves towards the direction of near-infrared zone, and the average light transmittance in the wavelength range of 400–800 nm ($T_{\mathrm{av}(400–800)}$) rises gradually at first and then declines markedly. The maximum $T_{\mathrm{av}(400–800)}$ in this group is 85.11% for the T40 C0.25 A9.5 T40 sample. Detailed transmittance numbers are presented in Table 1.

Figure 6b presents the light transmittance profiles for the films with PET/ZnS/Cu/Ag/ZnS structure. Different from the parameter adjustment rule for the TiO_2_ group, the ZnS-group samples were set with the constant thickness for the dielectric layers, only changing the parameters for the metallic layer. With the increase in the Ag layer thickness from 8 nm to 11 nm, the transmittance curves move integrally along the Y axis in the graph, revealing the similar feature of a parabolic pattern, and the general transmittance value increases first and then decreases. Insufficient layer thickness leads to the discontinuity of the metallic layer, which corresponds to an irregular interface, while an excess thickness will bring more light absorption, both scenarios are detrimental to the transmittance performance. The maximum value is 83.22% for the $T_{\mathrm{av}(400–800)}$ of the ZnS40 Cu0.25 Ag9.5 ZnS40 sample, while the ZnS40 Cu0.25 Ag11 ZnS40 sample possess the minimum $T_{\mathrm{av}(400–800)}$ of 76.47%, as listed in Table 1. The sample with a monolayered metallic layer shows a slightly smaller transmittance value than its counterpart with a Cu–Ag bilayer, probably ascribed to the inferior smoothness of the metallic layer, as seen in Figure 2a. The rougher layer interface will cause more scattering loss of light [12,15].

Figure 6c depicts the light transmittance patterns of the asymmetric PET/ZnS/Cu/Ag/TiO_2_ films. The sublayer thickness values of this group were designed based on the numerical simulation result. The thickness variation of the top layer was conducted to verify the optimum structure. The transmittance curves reveal a parabolic shape across the light wavelength from 350 nm to 1100 nm, with the maximum value at the wavelength around 600 nm for all the three samples. The $T_{\mathrm{av}(400–800)}$ value declines gradually from 85.12% to 83.15% when the thickness of the top layer increases from 31 nm to 45 nm, and the position of the transmittance curve moves towards the direction of near-infrared zone, which exhibits the consistent trend with that of the PET/TiO_2_/Cu/Ag/TiO_2_ group.

The comparison of the transmission properties among the representative samples from the three structure groups is illustrated in Figure 6d. The two samples with the TiO_2_ dielectric layer possess higher transmittance in the visible light range, while the sample with ZnS as both the dielectric layers presents better transmittance performance in the near-infrared range [27].

### 3.4. The Sheet Resistance of Multilayered TCFs

**Figure 7:**
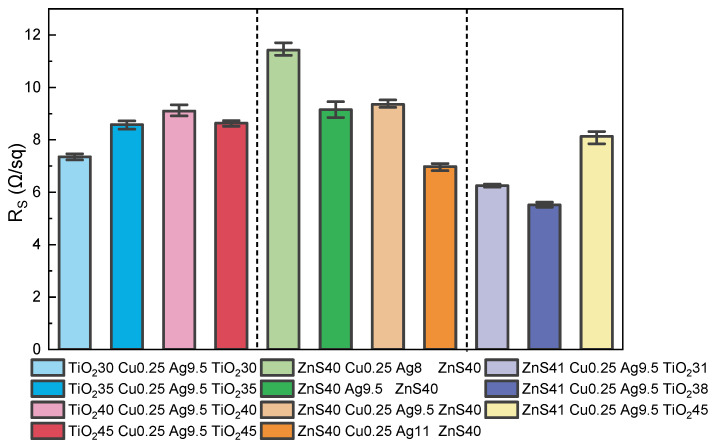
The sheet resistance results of multilayered TCFs.

The sheet resistance results of the abovementioned 11 multilayered TCF samples are presented for comparison, as shown in Figure 7. Table 1 lists the detailed data. In the group of PET/TiO_2_/Cu/Ag/TiO_2_ samples, the metallic layer remains a fixed structure parameter, the one in which the minimum thickness of dielectric layer possesses the smallest sheet resistance value. The other three samples with thicker dielectric layers have larger and relatively similar $R_{s}$ values, perhaps ascribed to the excessive impinging and perhaps thermal effect during the film growth of the top layer [22,33].

In the group of PET/ZnS/Cu/Ag/ZnS samples, the $R_{s}$ value drops significantly with the thickness increase in the metallic layer, which is consistent with the common rule for the effective cross-sectional area variation of a conductor. The one without a seed layer (ZnS40 Ag9.5 ZnS40) possesses a similar $R_{s}$ value to its counterpart with the Cu seed layer, perhaps due to the benefits of the excellent matching effect of the interface between ZnS and Ag layers [16].

Within the asymmetric PET/ZnS/Cu/Ag/TiO_2_ multilayer series, the sheet resistance exhibits a non-monotonic dependence on top-layer thickness. The optimal electrical performance, characterized by the minimum sheet resistance, is achieved at an TiO_2_ thickness of 38 nm. Considering the nominal metallic layer parameters are the same for this sample group, which accounts for the most conductive function, the variation of the $R_{s}$ could also be attributed to the influence of the top layer deposition process.

The asymmetric TCF group generally possesses the lowest sheet resistance among the three groups, with some values being even lower than that of nominal 11 nm thickness of the Ag layer (ZnS40 Cu0.25 Ag11 ZnS40). The excellent conductive performance is believed to arise from the suitable inner layer and interface status, which results from the mutual effects among the sublayers in the D/M/D structure.

### 3.5. Figure of Merit of the TCFs

The electrical conductivity and optical transmission performance of the multilayers are listed in Table 1. Besides direct numbers of the transmittance and sheet resistance, the figures of merit ($\phi_{\mathrm{TC}}$) [34] were calculated according to Equation (2):

(2) $$\phi_{\mathrm{TC}}=T^{10}/R_{s}$$

where T is the optical transmittance value (%).

The average figure of merit (FOM) value over the visible spectrum (400–800 nm), denoted as $\phi_{\mathrm{TC}(400–800)}$, serves as the primary metric for evaluating the electrical-optical performance of transparent conductive electrodes. Within the PET/TiO_2_/Cu/Ag/TiO_2_ group, the average FOM value changes very slightly when the dielectric layer thickness increases from 30 nm to 40 nm, revealing a good performance stability on a wide parameter scale. In multilayered PET/ZnS/Cu/Ag/ZnS architectures, $\phi_{\mathrm{TC}(400–800)}$ exhibits marked variation with the thickness modulation of the intermediate layers. Additionally, 9.5 nm seems to be the optimized thickness here, and the existence of a seed layer is beneficial to the comprehensive performance. The sample group with an asymmetric structure possesses the highest FOM number of the three. With suitable structure parameters, the PET/ZnS/Cu/Ag/TiO_2_ sample acquires both good light transmission and electrical conductivity on a flexible PET substrate with high surface roughness, being enhanced markedly on the performance compared with the counterparts with symmetric structures. The maximum $\phi_{\mathrm{TC}(400–800)}$ is 33.19 × 10^−3^ Ω^−1^ (ZnS41 Cu0.25 Ag9.5 TiO_2_38). The $\phi_{\mathrm{TC}(550)}$ number for each sample, corresponding to the property at the light wavelength of 550 nm, is also presented for reference, which is distinctly higher than the average value. However, the oscillatory transmittance profiles in Figure 5, attributable to light interference within the multilayer TCF stack [35], diminish the reliability of the single-wavelength $\phi_{\mathrm{TC}(550)}$ metric compared to the averaged FOM.

## 4. Discussion

### 4.1. The Optical Transmittance Comparison of the Simulation and Experimental Test

One of the main functions of dielectric layers is to improve the light transmittance for the D/M/D structured TCFs as anti-reflection films. It is highly effective to optimize the thickness of each sublayer in a TCF based on the interference theory of light. The optical designing software TFCalc (version number 3.8) is utilized to predict the light transmission and optimize the structure parameters for the flexible multilayered TCF on the PET substrate. After the model of the multilayered structure was built with specified layer materials, the complex index of refraction for the layer materials of TiO_2_, ZnS, Ag, Cu and PET [36,37,38] was adopted to simulate the transmittance of TCFs by the software program. The optimal result in the visible spectrum with the asymmetric structure is the scheme of ZnS41 Cu0.25 Ag9.5 TiO_2_38. The experiment result basically verifies the superiority of the optimal parameters obtained by numerical simulation, as seen in Figure 6c.

**Figure 8:**
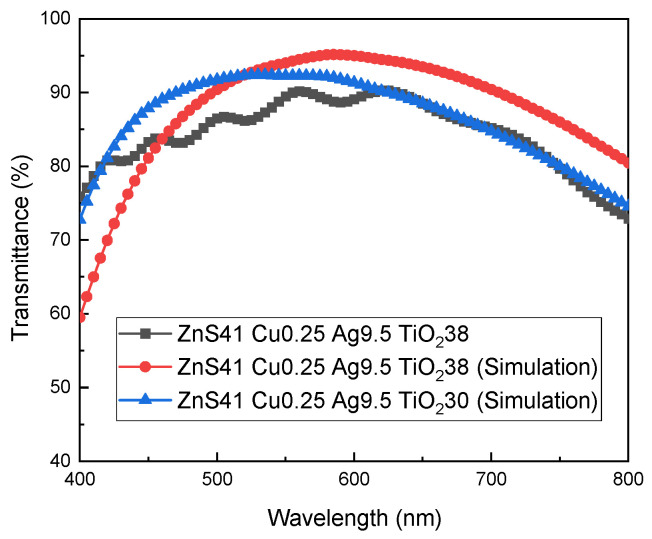
The transmittance curves of simulated and experimental PET/ZnS/Cu/Ag/TiO_2_ films.

The transmittance curves of the simulated and experimental results are demonstrated in Figure 8 for the structure of PET/ZnS/Cu/Ag/TiO_2_. For the sample of ZnS41 Cu0.25 Ag9.5 TiO_2_38, the transmittance values from both the experiment and simulation exhibit a similar trend in the visible light range, firstly increasing and then decreasing. However, the simulated curve moves towards the near-infrared region, unlike the test result. The $\phi_{\mathrm{TC}(400–800)}$ number of the simulation is 87.69%, larger than the experimental result of 84.40%. Based on the influence rule of dielectric layer thickness on the transmittance, as described in Figure 6a,c, the numerical simulation was conducted for the scheme of ZnS41 Cu0.25 Ag9.5 TiO_2_30 with a thinner top layer, aiming to better fit the tested transmittance curve. As shown in Figure 7, the simulation result of ZnS41 Cu0.25 Ag9.5 TiO_2_30 proves a better approximation to ZnS41 Cu0.25 Ag9.5 TiO_2_38 test result. The transmittance curves overlap each other in most regions of the visible spectrum, with close $\phi_{\mathrm{TC}(400–800)}$ numbers of 86.43% and 84.40%, respectively.

When proceeding with the optical simulation, an ideal status in the multilayered TCF is assumed, including the perfect uniformity and smoothness for each sublayer, interface and surface. However, the TCF sample prepared by magnetron sputtering on a high surface roughness substrate would deviate obviously from the ideal status, including features like irregular bonding between the interfaces, locally heterogeneous microstructure and fluctuated interface profile. These physical features of the sample were not taken into account during the numerical simulation and should be the main reason for the simulation deviation. The better fit between the simulation result and experimental data with a thinner top layer should be attributed to the smaller effective thickness for the sublayers, when depositing the TCF on the PET substrate with high surface roughness.

### 4.2. The Compatibility Between the Substrate and Sublayers of the Multilayered TCF

In the case of the physical vapor deposition (PVD) process, the ZnS particles generated from the magnetron sputtering possess distinctly higher energy than the vacuum evaporation process [8,39] (another popular preparation method for D/M/D structured TCFs). The high energy of the sputtered particle tends to form a film with the surface morphology comprised by large-sized clusters. It could be inferred that the covering of the PET substrate by the large clusters is more effective at alleviating the surface profile fluctuation inherited from the substrate. This phenomenon could be illustrated by Figure 1b,c, where the ZnS layer on the PET substrate exhibits a smaller surface roughness than the TiO_2_ layer. It is worth noting that the surface of the ZnS layer would possess a rougher morphology than the TiO_2_ layer when sputtering deposited on a highly smooth surface like electronic glass or PET with smoothing coating. Therefore, the ZnS bottom layer plays a role of more compatible partner with the high surface roughness substrate.

**Figure 9:**
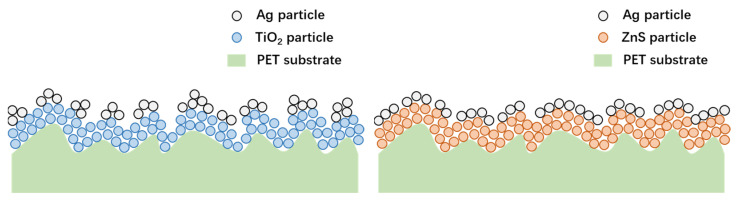
Illustration of the influence of bottom layer material on metallic layer forming.

Furthermore, the bottom layer of ZnS in the multilayered TCFs possesses excellent percolation property for the intermediate metallic layer compared with the other commonly used dielectric materials [16,20], which facilitates the metallic layer forming a film with better continuity, uniformity and smoothness at a very small thickness (usually ≤10 nm). For the intermediate monolayer of Ag, this phenomenon is well demonstrated by comparing Figure 2a and Figure 3a, based on which an illustration is presented in Figure 9. The Ag particles deposited on the TiO_2_ bottom layer reveal a stronger tendency to form isolated clusters, leading to a discontinuous metallic layer. Compared with the TiO_2_ layer, the bottom ZnS layer deposited on the high-surface-roughness PET substrate possesses a smoother surface. Further, the ZnS layer has a better percolation property for the intermediated Ag layer. Both factors contribute to the better surface quality of the PET/D/M structure with ZnS as the dielectric material. By comparing the surface morphology in Figure 2a,b and Figure 3a,b, it is evident that the metallic layers (with or without seed layer) possess better continuity when deposited on the ZnS bottom layer with high-surface-roughness PET substrate, where the advantage of adopting ZnS as the bottom dielectric is well demonstrated

During the PVD process, the growth of a metallic monolayer typically follows the Volmer–Weber model [23]. Firstly, the deposited atoms or particles form an isolated island-like structure, then the islands gradually grow larger, and the coalescence happens, afterwards the film grows by the mode of layer thickening. The introduction of the seed layer has proved the validity for facilitating the continuous metallic layer forming with a smaller critical thickness in a few cases [14,25,27,28]. For the multilayered samples in this study, the beneficial effect of the Cu seed layer is well demonstrated by comparing Figure 2a and Figure 2b, Figure 3a and Figure 3b, respectively. The Cu–Ag bilayers exhibit distinctly better performance in continuity and homogeneity than the Ag monolayer. This interfacial behavior originates from the distinct bond dissociation enthalpies (ΔH) of metallic pairs: $H_{Ag-Ag}=163\;kJ\cdot {mol}^{-1}$ versus $H_{Ag-Cu}=176\;kJ\cdot {mol}^{-1}$ [27]. The higher Ag-Cu binding energy drives preferential bonding during sputtering, thereby promoting interfacial homogeneity.

The top layer in the D/M/D structure not only behaves as the anti-reflection film, but also as the protective film to keep the TCF, especially the more active metallic layer, away from oxidation or other types of deterioration. The surface smoothness of the magnetron sputtered TiO_2_ layer is superior to the ZnS layer under the identical conditions, as seen in Figure 3d and Figure 4, which would reduce the light scattering loss at the surface. Besides the TiO_2_, deposited particles with greater sputtering power possess greater energy than that of ZnS, which may improve the uniformity and continuity of the metallic layer to obtain better electric conductivity and light transmittance. These factors could explain the superior combination property of PET/ZnS/Cu/Ag/TiO_2_ compared to PET/ZnS/Cu/Ag/ZnS, as listed in Table 1. Moreover, the TiO_2_ top layer does demonstrate better protective properties in the corresponding TCF samples. It was found that PET/TiO_2_/Cu/Ag/TiO_2_ and PET/ZnS/Cu/Ag/TiO_2_ configurations maintain stable electro-optical characteristics for several months under ambient conditions. Conversely, PET/ZnS/Cu/Ag/ZnS multilayers undergo accelerated degradation, exhibiting significant property deterioration within weeks. This divergency could be attributed to the dense and stable features of the top TiO_2_ layer, which well isolates the external reaction medium.

## 5. Conclusions

The flexible multilayered TCFs were deposited on the PET substrate with high surface roughness by magnetic sputtering, with ZnS, TiO_2_ as the dielectric layer materials and Ag, Cu as the metallic layer materials. The samples with an asymmetric PET/ZnS/Cu/Ag/TiO_2_ structure reveal superior optical and electrical performance compared to the symmetrical PET/TiO_2_/Cu/Ag/TiO_2_ and PET/ZnS/Cu/Ag/ZnS structures. The optimal values are $T_{\mathrm{av}(400–800)}$ 84.40%, $R_{s}$ 5.52 Ω/sq and $\phi_{\mathrm{TC}(400–800)}$ 33.19 × 10^−3^ Ω^−1^ for the light transmission and electrical conductivity properties of the visible light spectrum. The ZnS bottom layer acts more effectively in alleviating the fluctuation morphology inherited from the high-roughness substrate and facilitating the continuous film forming of the metallic layer. The Cu seed layer enhances the uniformity of the intermediate layers evidently. The TiO_2_ top layer behaves as the smoother and denser cap film compared with the ZnS layer, which provides the TCF with better light transmittance, electrical conductivity and chemical stability. The optimized structural parameters and reasonable compatibilities between the substrate and sublayers in the flexible asymmetric TCF should account for its satisfactory combination properties.

## Figures and Tables

**Table 1:** The optic and electric performance of TCFs.

TCF	$R_{s}$ (Ω/sq)	$T_{550}$ (%)	$T_{\mathrm{av}(400–800)}$ (%)	$\phi_{\mathrm{TC}(550)}$ (10^−3^ Ω^−1^)	$\phi_{\mathrm{TC}(400–800)}$ (10^−3^ Ω^−1^)
TiO_2_30 Cu0.25 Ag9.5 TiO_2_30	7.35	86.43	83.12	31.64	21.42
TiO_2_35 Cu0.25 Ag9.5 TiO_2_35	8.58	86.00	84.65	25.79	22.02
TiO_2_40 Cu0.25 Ag9.5 TiO_2_40	9.10	88.18	85.11	31.23	21.91
TiO_2_45 Cu0.25 Ag9.5 TiO_2_45	8.64	89.29	82.88	37.30	17.70
ZnS40 Cu0.25 Ag8 ZnS40	11.42	81.54	79.98	11.37	9.37
ZnS40 Ag9.5 ZnS40	9.15	85.08	82.54	21.71	16.04
ZnS40 Cu0.25 Ag9.5 ZnS40	9.35	83.39	83.22	17.39	17.05
ZnS40 Cu0.25 Ag11 ZnS40	6.98	79.89	76.47	15.18	9.79
ZnS41 Cu0.25 Ag9.5 TiO_2_31	6.25	91.11	85.12	63.03	31.93
ZnS41 Cu0.25 Ag9.5 TiO_2_38	5.52	89.54	84.40	59.97	33.19
ZnS41 Cu0.25 Ag9.5 TiO_2_45	8.13	86.10	83.15	27.52	19.42

## Data Availability

The original contributions presented in this study are included in the article. Further inquiries can be directed to the corresponding authors.
